# Current therapies and progress in the treatment of advanced gastric cancer

**DOI:** 10.3389/fonc.2024.1327055

**Published:** 2024-02-26

**Authors:** Hongyu Li, Ming Shen, Shihao Wang

**Affiliations:** Department of Gastroenterology, The People’s Hospital Of Changxing Country, Zhejiang, China

**Keywords:** advanced gastric cancer, immunotherapy, anti-angiogenic therapy, immune checkpoint inhibitors, treatment

## Abstract

Gastric cancer (GC) remains one of the most life-threatening disease worldwide with poor prognosis because of the absence of effective treatment and the delay in diagnosis. Due to the delay of diagnosis, a large proportion of GC patients are diagnosed as advanced GC, with extreme short lifespan. In the past few years, some pivotal progress and novel therapies was proposed, and conducted into clinical researches and practice. In this study, we summarized the development of several novel immunotherapy or targeted treatment modalities for advanced GC, including immune checkpoint inhibitors, anti-angiogenic therapy and cancer vaccines. Additionally, the advantage and potential weakness in each of these therapeutic methods are also listed. Finally, we discussed the promising research direction of advanced GC treatment, and the limitation in basic and clinical research of advanced GC, including the combination of immunotherapy and targeted therapy.

## 1 Introduction

Gastric cancer (GC) is the fourth leading cause of cancer-related deaths worldwide and the fifth most frequently diagnosed malignancy (1). South American and Asian nations account for the majority of new diagnoses of stomach cancer each year (2). Patients with advanced GC have a poor prognosis and a short lifespan of roughly one year because of the absence of effective medications and the delay in detection (3). Radiation therapy, chemotherapy, and targeted therapy are the treatments that are most frequently utilized, while primary criteria used to establish a treatment plan are the illness stage, the existence of biomarkers, and the recommended regimen of the treating physician (2). For the treatment of advanced gastric cancer, medications such regorafenib, imatinib, entrectinib and Larotrectinib are frequently utilized (3–5). Traditional treatments, however, in many cases cause multi-drug resistance and tumor relapse (6). The most recent 8th edition of the American Joint Committee on Cancer (AJCC) cancer staging system (cTNM), which was released in 2017, has significantly improved decisions on the treatment of GC (7). Despite the fact that the many classification systems and terminology used for this disease around the world can make it difficult to diagnose GC based on subtypes, it is obvious that it still remains a fatal disease that is not meaningfully controlled by current treatment options or earlier detection strategies (2). Due to its tremendous potential, immunotherapy has recently emerged as a revolutionary therapy for treating advanced GC and has attracted the interest of researchers everywhere.

Studies have indicated that the development of immune check point inhibitors (ICIs), such as antibodies against the cytotoxic T-lymphocyte antigen CTLA-4 (8), targeted immunotherapies in the pathways of programmed cell death 1 (PD-1) and programmed death-ligand 1 (PD-L1) antibodies, have transformed the treatment paradigms of a variety of solid tumors by efficiently killing cancer cells through activation of the immune response (8).

ICIs have already shown efficacy and safety in clinical trials for several cancers. In order to treat advanced gastric tumors, a number of ICIs, including pembrolizumab, avelumab, sintilimab, tislelizumab, and ipilimumab, have been given clinical approval (3, 9–11). The outcomes of recent trials testing these novel drugs raise the question of how to identify the people who might benefit the most. Therefore, advances in our understanding of the biology and mechanisms behind various clinical characteristics of the disease will make new drug development possible (8). Although chemotherapy is still the mainstay of treatment for patients with advanced gastric cancer, progress in its molecular characterization and the creation of tailored medicines may represent a promising strategy (12). In this study, we sought to examine the perspective and development of several immunotherapy treatment modalities for GC. Additionally, we discussed the difficulties that immunotherapies now face as well as potential solutions to these problems, such the combination of immunotherapy and targeted therapy.

## 2 Molecular profile

**Figure 1:**
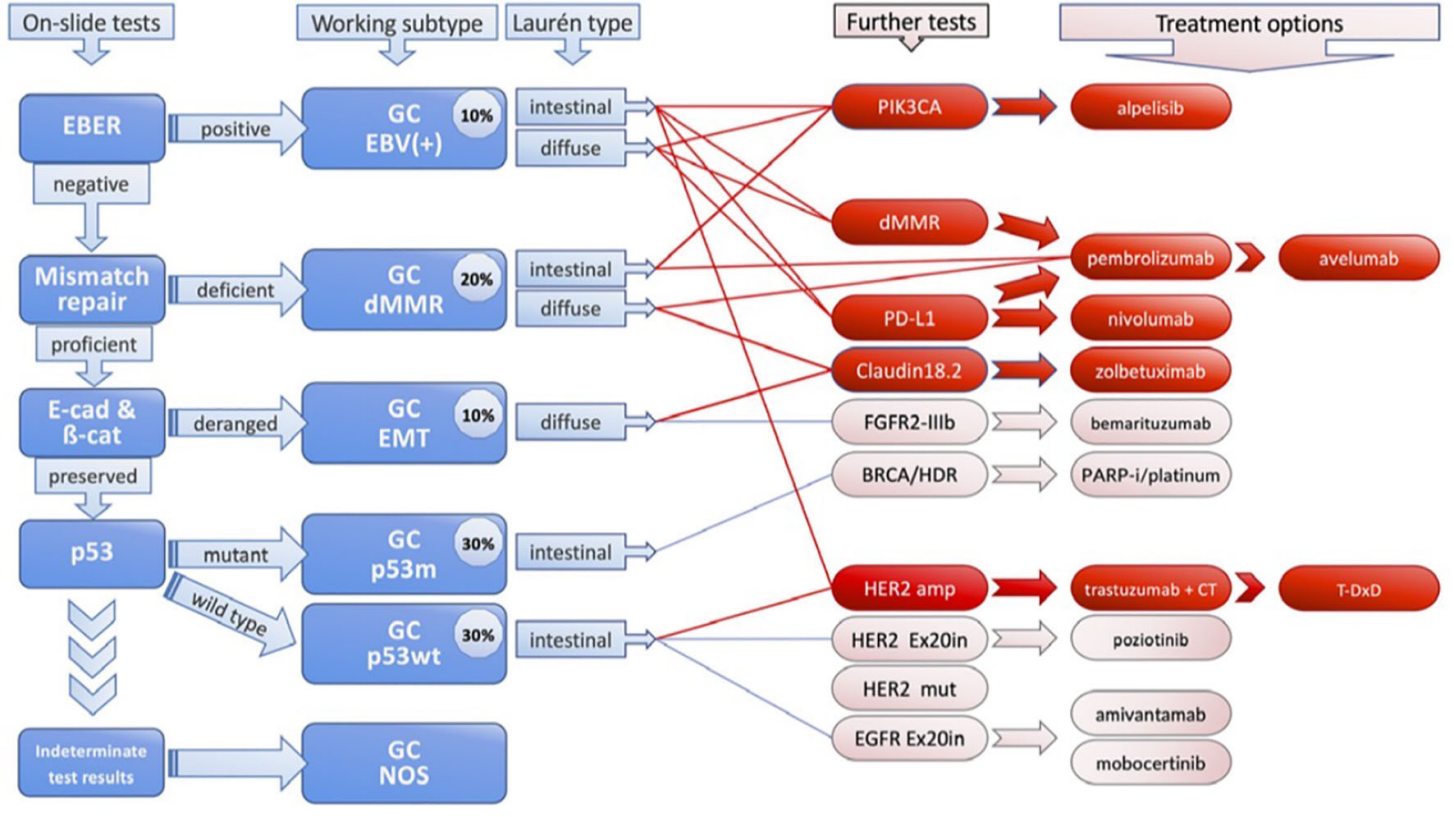
Molecular Classification of Gastric Cancer.

The organization of GC is gradually evolving away from histological classification and toward more complicated molecular categorization (8, 13, 14). Lauren classification (1960s) and the WHO classification (2010) are most commonly used in GC classification (15). Lauren Classification system divided GC into three main subgroups using the structural cellular components of the disease: well differentiated (non-cardia/intestinal), poorly differentiated (cardia/diffuse), and mixed type disease (**Figure 1**). A fourth new subtype, solid GC, is also included (2, 16). While, tubular, papillary, poorly cohesive and mucinous are subtypes in WHO classification (15). The distribution of subtypes varies greatly by region, and more significantly, the clinicopathological features of gastric cancer are evolving, with a declining prevalence of distal, well differentiated type tumors and a rising proportion of poorly differentiated/diffuse histology (17). However, these classified subtypes have shown minimal relevance in clinical practice because they lack predictive value and have minimum therapeutic implications.

Scientists have only recently started to fully understand the true heterogeneity of gastric cancer (GC), and they have been the pioneers in presenting its molecular characterization (18). They found that amplifications in the genes encoding for receptor tyrosine kinase proteins (RTKs) including VEGFA, ERBB2 (also known as HER2), EGFR, cell-cycle mediators, JAK2, FGFR2, ERBB3, and KRAS or NRAS are present in about 40% of these tumors (19). By demonstrating these targetable molecular traits, the majority of phase II and III clinical trials for GC during the subsequent decade considered treatments by targeting these molecular abnormalities (20). In 2014, using six molecular platforms (whole-exome sequencing, messenger RNA sequencing, array-based somatic copy number analysis, microRNA sequencing, array-based DNA methylation profiling and reverse-phase protein array), clustering analysis of data from 295 GC patient samples from around the world was carried out as part of The Cancer Genome Atlas (TCGA) program (17). The analysis identified four subtypes: microsatellite instability (MSI), chromosomal instability (CIN), Epstein-Barr virus (EBV) and genomically stable (GS) (21). Although GC is classified molecularly, these findings have not yet been applied in therapeutic settings (8). Moreover, an analysis of over 1000 gastric cancer samples revealed that non-Asian tumors had higher expression of T cell markers (CD45R0, CD3 and CD8), including CTLA-4 signaling, and lower expression of the immunosuppressive T regulatory cell marker FOXP3 compared to Asian tumors. This data suggests that disparities in immunological profiles merit additional exploration, as does a comparison of ICI response between Asian and non-Asian populations (9).

## 3 Immunotherapy treatments for advanced GC

### 3.1 Immune checkpoint inhibitors (ICIs)

**Figure 2:**
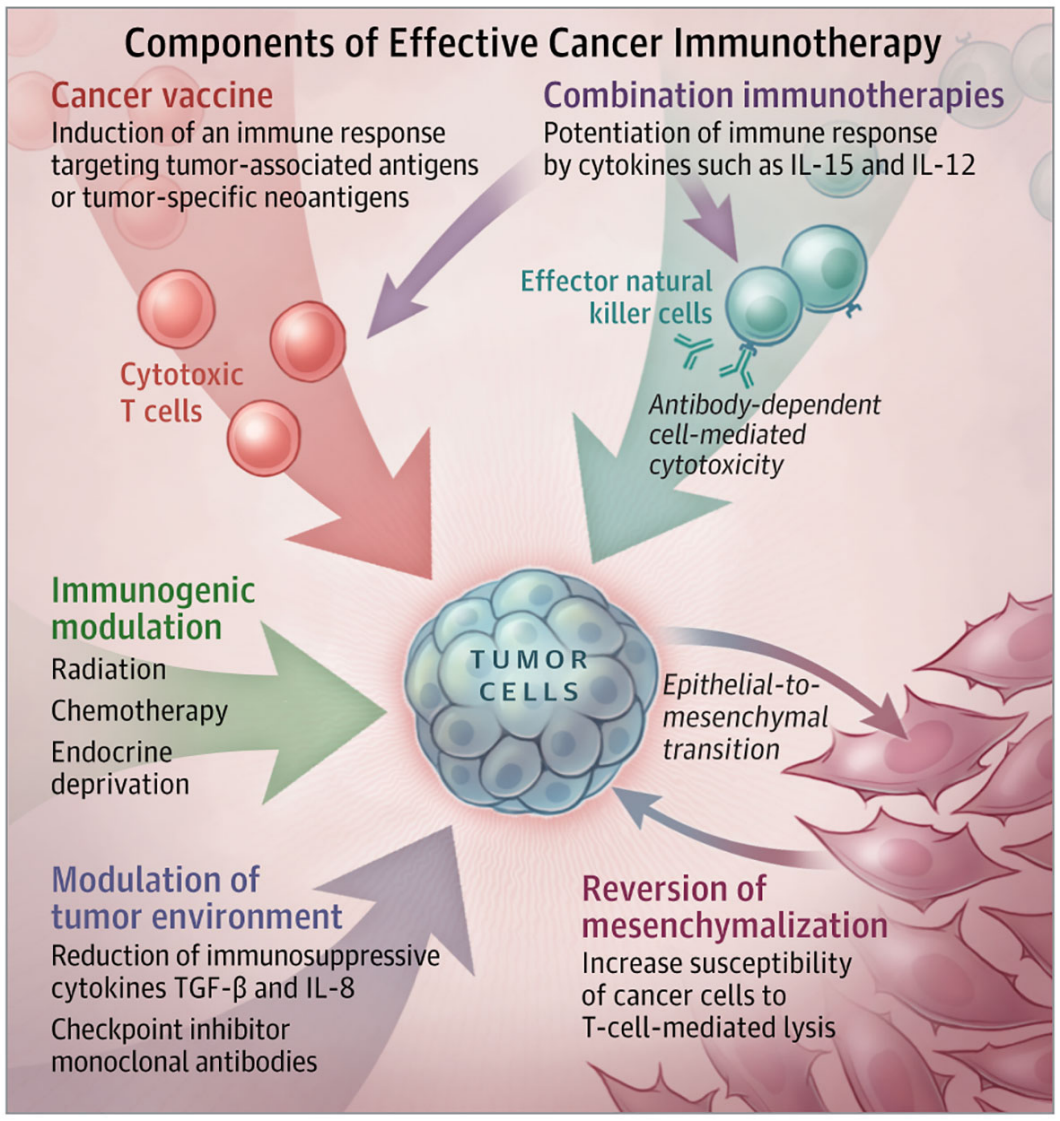
Components of effective immunotherapy in gastric cancer.

Over the past few years, a better understanding of the molecular mechanism of gastric cancer has greatly facilitated the development of novel therapies (22). Immunomodulating drugs are actively reshaping the medical field in a variety of cancer types and represent a potential path for GC (23) (**Figure 2**). Tumor cells may exploit immunological checkpoints in an inadvertent manner to avoid host immunosurveillance and immune destruction (24). Immune checkpoints may be counteractively used by tumor cells to evade host immunosurveillance and escape immune destruction. Because of that, inhibition of checkpoints by ICIs helps restore host immunity against tumor cells (25, 26). ICIs could successfully disrupt immune checkpoint interactions, resulting to tumor cell death by activation of the host immune system (27).

Individual patients with advanced-stage cancer have shown durable responses to ICI treatment. As a result, these agents represent the most promising new therapy alternatives for GC patients (24). Anti-PD-L1 (avelumab), anti-PD-1 (nivolumab, pembrolizumab) and as well as anti-CTLA-4 (ipilimumab, tremelimumab), have shown enhanced results in cancer patients. Recent analyses have also proven the efficacy of these medicines in GC patients (8). Ipilimumab was the first ICI approved in the world (2011) to treat melanoma. Immunotherapies have since transformed advanced gastric cancer therapy techniques. There are mainly three types of ICIs, anti-PD1/PD- L1 and anti-CTLA4 antibodies. Inhibitors targeting these immune checkpoints have been generated and studied in pre-clinical and clinical trials (3). These achievements in immunotherapy have marked a new era for advanced gastric cancer treatment (3, 28). Indeed, treatment with ICIs has elicited sustained responses in individual patients with advanced- stage cancer. Thus, these agents constitute the most promising new therapeutic options for patients with GC (24, 29).

### 3.2 PD-1 inhibitor

The primary functions of immune checkpoint inhibitor (known as PD-1) is regulating the alterations caused to the cellular system, this is a promising cancer therapeutic target in different cancers including gastric cancer. Typically, immune checkpoint inhibitors target key regulatory molecules associated with escaping or protective processes for tumor cells from immune attack. Thus, PD-1 is a prominent concern in the regulation of cytotoxic activity of anti-tumor T cells. A wide range of studies based on extensive randomized controlled trials have concluded that gastric cancer and associated cancer types can achieve disease control and improve overall survival from treatment with PD-1 inhibitors treatment (30). Nivolumab, a PD-1 inhibitor, is a monoclonal antibody that was approved by the FDA in 2014 for the treatment of advanced gastric cancer (31). Through phase III clinical studies, which were carried out in more than 40 Asian nations, the benefits of nivolumab against advanced GC were investigated (3). According to preliminary findings, nivolumab might considerably improve patient survival compared to placebo. Nivolumab therapy in patients with GC showed 12-month overall survival rates of 26.2% compared to 10.9% with placebo treatment, suggesting a hopeful cure for this population with a dismal prognosis (31). In particular, nivolumab has received approval for use in clinical settings as a cutting-edge strategy to treat advanced and recurring GC (2).

Another effective PD-1 inhibitor is pembrolizumab. Pembrolizumab was most recently given expedited clearance by the Food and Drug Administration (FDA) in conjunction with trastuzumab, first-line chemotherapy for patients with HER2 positive advance GC, based on the interim findings of KEYNOTE-811 (9). To compare the effectiveness of pembrolizumab, pembrolizumab plus chemotherapy, or chemotherapy a total of 763 patients were randomly assigned. Pembrolizumab was shown to be noninferior to chemotherapy in patients with untreated, advanced GC, with less adverse effects in this phase 3 randomized clinical trial. However, pembrolizumab or pembrolizumab with chemotherapy did not outperform treatment in terms of overall survival (OS) or progression-free survival (PFS) (32).

### 3.3 PD-L1 inhibitors

PD-L1 is a cancer cell surface marker that is overexpressed on multiple cancer cells and escapes immune system identification by suppressing T-cell inhibition (33). Prevention of GC with the PD-L1 inhibitor Pembrolizumab (Keytruda) has been authorized for third-line usage in gastric cancer based on the results of the phase II KEYNOTE-059 trial, which boosted the rate of responses to 11.6% compared to 2.3% in the control arm (2). Avelumab, durvalumab, and atezolizumab are a few of the well-known PD-L1 inhibitors (3). A phase III trial of the anti-PD-L1 mAb avelumab in people with advanced gastric cancer showed that it was well tolerated. Patients from Japan who took avelumab showed significant rates of overall response and survival. Additionally, avelumab’s effectiveness against advanced gastric cancer is increased when used in conjunction with other treatments (12). The mechanism by which PD-L1 inhibitors contribute to advanced gastric cancer may be that PD-L1 inhibition activates DC cells, T lymphocytes, and natural killer cells, resulting in gastric tumor elimination (3). It appeared that high levels of PD-L1 (an adaptive immune resistance-type mechanism) were related with CD8+ T-cell infiltration in GC, pointing to the potential effectiveness of anti-PD-1/PD-L (8)However, further research is required to fully comprehend the prognostic significance of immune cell activity and PD-L1 expression. Interesting new findings demonstrated that patients with greater CD8+ T-cell density have higher PD-L1 expression and poorer outcomes in a small cohort of resected GCs (23).

### 3.4 CTLA-4 Inhibitors

CTLA-4 is an essential component of the human immune system. Because CTLA-4 is identical to CD28, it can control or even block CD28 signaling (3). CTLA-4 is a well-studied immunological checkpoint in GC. However, the predictive impact of CTLA-4 expression in GC is unclear (34).

CTLA-4 inhibitors tremelimumab and ipilimumab have been evaluated in clinical trials of advanced gastric cancer (3). Combination therapy of ipilimumab and nivolumab has been approved to treat advanced gastric cancer. However, the efficacy of CTLA-4 inhibitor as a monotherapy in advanced gastric cancer remains to be further investigated (3).

## 4 Anti-angiogenic therapy

Angiogenesis is a well-known phenomenon defined as the process of new blood vessels formation. It is a complex and dynamic process, which contributes crucially to tumor growth, invasion, and metastasis. This process is regulated by various pro- and anti-angiogenic molecules involved in the progression and development of cancer. Researchers have demonstrated the molecular processes associated with tumor angiogenesis. Most prominent biomolecules elucidated by advances in molecular and cellular biology in angiogenesis, include growth factors, chemokines, and adhesion factors. Based on these molecules, targeted therapeutic research has driven treatment with anti-angiogenic agents to become a promising therapeutic strategy against different cancer types including gastric cancer. Some of the most common and prominent anti-angiogenic agents are tyrosine kinase inhibitors and monoclonal antibodies, which can target vascular endothelial growth factor pathway (35). Angiogenesis is a promising therapeutic target which plays a key role in cancer cell proliferation and metastasis. Several studies indicated that pharmacologic blockade of angiogenesis may be a promising therapeutic approach. In the several clinical trials different anti-angiogenic therapies for gastric cancer, including anti-VEGF or anti-VEGF receptor (VEGFR)-2 monoclonal antibodies, VEGF-Trap and VEGFR tyrosine kinase inhibitors, the anti-VEGFR-2 antibody ramucirumab was demonstrated to prolong overall survival both as a single agent and in combination with paclitaxel as a second-line chemotherapy (36–39). The next step in anti-angiogenic therapy is to evaluate anti-angiogenic therapy in combination with immune checkpoint inhibitors, assess the safety and efficacy of combination therapy with chemotherapeutic agents as an earlier treatment option or in the perioperative setting, and establish a clinically meaningful biomarker. Ramucirumab’s early results in combination with anti-PD-1/PD-L1 therapy are promising for the continued development of gastric cancer treatments to increase patient survival (39, 40).

## 5 Conclusion

Development of immunotherapy in advanced gastric cancer has demonstrated great advantages over traditional therapies. However, there still exists various challenges that have severely limited the clinical application of immunotherapy in advanced gastric cancer, for instance, the side effects and toxicity of ICIs, cancer vaccines and CAR-T therapies.

## Author contributions

SM: Conceptualization, Investigation, Methodology, Validation, Writing – original draft. HL: Data curation, Formal Analysis, Investigation, Resources, Visualization, Writing – review & editing. SW: Conceptualization, Methodology, Project administration, Supervision, Writing – original draft, Writing – review & editing.
